# Genetic Mapping of a Light-Dependent Lesion Mimic Mutant Reveals the Function of Coproporphyrinogen III Oxidase Homolog in Soybean

**DOI:** 10.3389/fpls.2020.00557

**Published:** 2020-05-08

**Authors:** Jingjing Ma, Suxin Yang, Dongmei Wang, Kuanqiang Tang, Xing Xing Feng, Xian Zhong Feng

**Affiliations:** ^1^Key Laboratory of Soybean Molecular Design Breeding, Northeast Institute of Geography and Agroecology, The Innovative Academy of Seed Design, Chinese Academy of Sciences, Changchun, China; ^2^University of Chinese Academy of Sciences, Beijing, China

**Keywords:** *Glycine max*, *lesion mimic mutant 2*, tetrapyrrole biosynthesis, coproporphyrinogen III oxidase, pathogen resistance

## Abstract

Lesion mimic mutants provide ideal genetic materials for elucidating the molecular mechanism of cell death and disease resistance. Here, we isolated a *Glycine max lesion mimic mutant 2-1* (*Gmlmm2-1*), which displayed a light-dependent cell death phenotype. Map-based cloning revealed that *GmLMM2* encods a coproporphyrinogen III oxidase and participates in tetrapyrrole biosynthesis. Knockout of *GmLMM2* led to necrotic spots on developing leaves of CRISPR/Cas9 induced mutants. The *GmLMM2* defect decreased the chlorophyll content by disrupting tetrapyrrole biosynthesis and enhanced resistance to *Phytophthora sojae*. These results suggested that *GmLMM2* gene played an important role in the biosynthesis of tetrapyrrole and light-dependent defense in soybeans.

## Introduction

Plants have developed sophisticated defense mechanisms to protect against attack by different pathogens. The hypersensitive response (HR) is an effective resistance reaction, which induces rapid programmed cell death (PCD) in infected areas to inhibit further invasion of pathogens in normal cells (Dangl et al., 1996). HR-mediated PCD is accompanied by a burst of reactive oxygen species (ROS), triggered by the expression of pathogenesis-related (PR) genes (Qiao et al., 2010; Dickman and Fluhr, 2013). Lesion mimic mutants (*lmms*) are plants that spontaneously develop necrotic lesions caused by cell death, without any pathogen infection or abiotic stress, which are similar to disease symptoms or HR. Therefore, *lmms* are powerful tools for studying the mechanisms of HR, PCD, and disease resistance. Earlier studies showed that a number of *lmms* plants present enhanced disease resistance compared with their progenitor parents. Subsequently, a number of *LMMs* genes were mapped and cloned, and major pathways were clarified, including chlorophyll synthesis, fatty acid and lipid biosynthesis, secondary messenger, and kinase signaling pathways (Hoisington et al., 1982; Wolter et al., 1993; Takahashi et al., 1999; Lorrain et al., 2003; Tsunezuka et al., 2005; Moeder and Yoshioka, 2008; Bruggeman et al., 2015; Sun et al., 2017).

The tetrapyrrole biosynthetic pathway is a multi-branched pathway. The most of the intermediate molecules of the tetrapyrrole biosynthetic pathways are photosensitizers. Under the light, these highly photoreactive sensitizers potentially generate singlet oxygen that lead to the accumulation of copious amounts of ROS and the subsequent formation of photodynamic damage and spontaneous necrotic spots on developing leaves (Ishikawa et al., 2001). The reduced activity of some tetrapyrrole biosynthetic enzymes lead to the accumulation of photosensitized intermediates and cell death in leaves. For example, defects in uroporphyrinogen III decarboxylase (UROD) in the maize *lesion mimic22* (*Les22*) mutant, porphobilinogen deaminase (*PBGD*) in the maize *camouflage1* (*cf1*) mutant and Arabidopsis *rugosa1* (*rug1*) mutant, and 5-aminolevulinic acid dehydratase (ALAD) in the cotton *Ghlmm* mutant induce spontaneous cell death in leaves (Hu et al., 1998; Huang et al., 2009; Quesada et al., 2013; Chai et al., 2017).

Many *lmm*s exhibited up-regulation of resistance-related genes and enhanced pathogen resistance (Takahashi et al., 1999; Guo et al., 2013; Sathe et al., 2019). *PR1* (*Pathogensis-Related1*) and *NPR1* (*NON-EXPRESSOR OF PATHOGENESIS-RELATED GENES1*) are strongly correlated with the onset of systemic acquired resistance (SAR) (Weigel et al., 2001; van Verk et al., 2008; Zheng and Dong, 2013). Endogenous signal molecules such as jasmonic acid (JA) and salicylic acid (SA) play an important role in induced resistance against pathogen infection. The transcript levels of SA-responsive marker genes, *GmPR10* (*Pathogensis-Related protein10*) and *GmSnRK1.1* (*Sucrose Non-fermenting-1-Related Protein Kinase1*) were significantly increased following infection with *P. sojae* (Xu et al., 2014; Wang et al., 2019). *AOS* (*Allene Oxide Synthase*) generate precursors of the hormone JA, and *COI1* (*Coronatine Insensitive 1*) is a critical component of the JA receptor. Both AOS and COI1 play an important role in JA mediated defense (Xie et al., 1998). It was also reported that *OsJAMyb* (*Jasmine Acid induced MYB*) enhanced blast resistance in transgenic rice (Cao et al., 2015).

Coproporphyrinogen III oxidase (CPO) is an enzyme in the tetrapyrrole biosynthetic pathway that catalyzes coproporphyrinogen III (Coprogen III) to protoporphyrinogen IX (Proto IX) via the oxidative decarboxylation of two propionate groups of the side chains of pyrrole rings A and B to vinyl groups (Tanaka et al., 2011). Two unrelated structural and functional CPOs, CPO/HemF and CPO/HemF, catalyze the reaction under aerobic and anaerobic conditions, respectively. CPO/HemF belongs to a family of monooxygenases and CPO/HemN belongs to the radical S-adenosylmethionine family (Ouchane et al., 2004; Phillips et al., 2004). CPO/HemF exist widely in living organisms, from lower forms of life and higher living organisms. The mutations in human CPO/HemF associated with many of the disease hereditary coproporphyria. The crystal structure of human CPO/HemF reveals critical residues for catalytic activity and substance binding sites. Disruption of CPO/HemF causes necrotic spot formation in *Nicotiana tabacum*, *Arabidopsis*, and rice (Kruse et al., 1995; Ishikawa et al., 2001; Sun et al., 2011; Wang et al., 2015).

In the present study, we carried out map-based cloning of the *Glycine max lesion mimic mutant 2-1* (*Gmlmm2-1*) in soybean and confirmed *GmLMM2* gene encodes CPO/HemF and its protein located in the chloroplast. Suppression of GmLMM2 disrupts the chloroplast structure and tetrapyrrole synthesis pathway. The lesion mimic phenotypes in *Gmlmm2-1* depend on the light and might help to enhance pathogens resistance to *Phytophthora sojae*. Our results suggest that GmLMM2 plays an important role in the biosynthesis of tetrapyrrole and immunity in soybean.

## Materials and Methods

### Plant Materials and Leaf Staining

The soybean cultivar “Williams 82” and “Hedou 12” were obtained from the Chinese Academy of Agricultural Sciences (Cheng et al., 2016). The *Gmlmm2-1* mutant was isolated from an M_2_ population induced by ethyl methane sulfonate (EMS) (Feng et al., 2019). For further analysis, the *Gmlmm2-1* mutant was backcrossed to “Williams 82” four times to purify the *GmLMM2* mutation. All the plants were grown in Changchun, China. The *Columbia* (Col-0) ecotype of *A. thaliana* plants were grown in a growth chamber (Percival, IA, United States) under 150 μmol m^–2^s^–1^ 16 h light/8 h dark cycles at 25°C.

The first compound leaf of the 15-day-old “Williams 82” and the *Gmlmm2-1* mutant were used in a half-leaf shading experiment. Small pieces of aluminum foil were used to cover half of the *Gmlmm2-1* mutant and “Williams 82” leaves to prevent light exposure; 5 days later the *Gmlmm2-1* mutant and “Williams 82” leaves were photographed and staining with different reagents. Leaf trypan blue (TB) staining was performed as the method described by Yin et al. (2000). H_2_O_2_ accumulation was detected by 3, 3’-diaminobenzidine (DAB) staining (Thordal-Christensen et al., 1997).

### Determination of Pigment Content, Gas Exchange, and Chloroplast Fluorescence

To determine pigment content, leaves of 3-week-old *Gmlmm2-1* mutant and “Williams 82” were collected and measured as previously described (Lichtenthaler and Wellburn, 1983). Net photosynthetic rate (Pn) and transpiration rate (Tr) of leaves were measured using Li-6400 photosynthesis equipment (Li-Cor, Lincoln, NE, United States) (Yamori et al., 2011). F_0_ (initial fluorescence) and Fm (maximal fluorescence) values were measured using a chlorophyll fluorometer OS-30p (Opti-Sciences, Hudson, NY, United States). Fv/Fm (maximum quantum efficiency of photosystem II) and Fv/F_0_ (maximum primary yield of photochemistry of PSII) were calculated as previously described (Genty et al., 1989). All measurements were made using three plants from 10:00 am to 12:00 noon during the R1 period with three biological replicates.

### Measurement of Malonyldialdehyde, Hydrogen Peroxide Contents and Chlorophyll Precursor Levels

Malonyldialdehyde (MDA) levels in leaves were measured as described by Hodges et al. (1999). H_2_O_2_ content was quantified using an H_2_O_2_ detection kit according to the manufacturer’s instructions (Comin Biotechnology, Suzhou, China). The absorbance of yellow compound was measured at 415 nm using a NanoPhotometer (Implen).

Coprogen III content was measured as described by Pratibha et al. (2017). The absorbance of Coprogen III was measured at a wavelength of 402 nm. Uroporphyrinogen III (Urogen III) content was determined as described by Bogorad (1962). The absorbance of Urogen III was measured at a wavelength of 405 nm. The Proto IX, Mg-protoporphyrin IX (Mg-proto IX) and protochlorophyllide (Pchlide) contents were measured as described by Rebeiz et al. (1975). All above measurements were obtained from three biological replicates.

### Transmission Electron Microscopy and Subcellular Localization Assays

True leaves were cut into smaller sections of approximately 1 × 1 mm, and placed in 1.5 mL fixation solution (2.5% glutaraldehyde with phosphate, pH 7.2), and vacuumed until completely immersed in the solution. The samples were subsequently fixed with 1% osmium tetroxide at 4°C. Samples were then treated according to previously described methods (Kowalewska et al., 2016). The samples were sliced to 70 nm thickness with a MT-X (RMC, Tucson, AZ, United States) ultramicrotome and stained with uranyl acetate and lead citrate. The samples were observed using a Hitachi H-7650 electron microscope (Tokyo, Japan).

To determine the subcellular location of GmLMM2, a full-length cDNA fragment of *GmLMM2* was amplified using the primer pairs listed in Supplementary Table S1 and inserted into the modified expression vector pUC19-GFP (Zheng et al., 2016). The pFL1004 construct (Supplementary Figure S8) was introduced into Arabidopsis (Col-0) mesophyll protoplasts using 20% polyethylene glycol (Meyer et al., 2006). Fluorescence was monitored using a confocal laser scanning microscope Leica SP8 (Leica, Solms, Germany). We detected the fluorescence of GFP wavelengths at 488 nm (excitation) and 495–540 nm (emission) and chlorophyll auto fluorescence wavelengths at 488 nm (excitation) and 680–700 nm (emission).

### Genetic Mapping and Phylogenetic Analysis

The INDEL markers for preliminary mapping were same as described previously (Song et al., 2015). New molecular markers for fine mapping are listed in Supplementary Table S1. The *Gmlmm2-1* mutant was re-sequenced with a depth of approximately 30× using illumina Hiseq2000 (Song et al., 2015). The Genome Analysis Toolkit (GATK, version 3.8) was used to detect single nucleotide polymorphisms (SNPs; McKenna et al., 2010). Sequences were deposited at the National Center for Biotechnology Information (NCBI) under the accession number SRP149750.

The amino acid sequences of CPOs/HemF were downloaded from Phytozome^1^, Phytozome V12.0. CPOs/HemF sequences were aligned with DNAMAN 8.0. MEGA7^2^ and used to build a phylogenetic tree via the maximum likelyhood method with 1000 bootstrap replications. CPOs/HemF motifs were analyzed using MEME^3^ and TBtools software (Bailey et al., 2015). Synteny was analyzed using MCScanX (Wang et al., 2012).

### Plasmid Construction and Soybean Transformation

For mutation complementation, the 5,374 bp genomic DNA fragment of *GmLMM2* (including 2,008 bp promoter region and 3,366 bp gene region) was inserted into the pCAMBIA3301 vector between *Sac* I and *Bam* HI sites. The resultant pFL1002 plasmid (Supplementary Figure S6) was then transformed into the cotyledonary explants of the *Gmlmm2* mutant via *Agrobacterium-*mediated transformation (Zhao et al., 2016).

To knockout the *GmLMM2* gene in the ‘DongNong50 (DN50)’ soybean cultivar, the modified pSC1-Cas9 was used as described by Du et al. (2016). A 20-nt single guide RNA (sgRNA) was identified using the web-based tools CRISPR-P^4^ and CRISPR-PLANT^5^, which targeted 195 to 214 position in the first exon of GMLMM2. The oligo pairs corresponding the sgRNA were annealed and ligated into a plasmid pSC1-Cas9 digesting with BspQ I. The sgRNA expression was driven under GmU6-16g-1 promoter in the recombinant plasmid pFL1003. The recombinant plasmid pFL1003 (Supplementary Figure S7) was transformed into DN50 as above (Zhao et al., 2016). The primers used for the construct are listed in Supplementary Table S1.

### RNA Isolation and Real-Time Quantitative PCR

Total RNA was isolated from different soybean tissues using TRIzol (Invitrogen, Carlsbad, CA, United States), and genomic DNA was digested with DNase I (Takara Biotechnology, Dalian, China). cDNA was synthesized using a Fast Quant RT Kit (TIANGEN, Beijing, China). RT-qPCR was performed in a Gene Amp 5700 sequence Detection System with SYBR^®^ premix Ex Taq^TM^ regent (Takara). *GmActin11* was used as the reference gene (Zeng et al., 2017), the data were analyzed using the 2^–ΔΔCT^ method (Livak and Schmittgen, 2001). Three biological replicates, each with three technical replicates, were performed. The primers used in RT-qPCR are listed in Supplementary Table S1.

### Pathogen Inoculation

*Phytophthora sojae* strain P7076 (*P. sojae* P7076) was cultivated at 25°C on 10% (v/v) V8 juice agar in a polystyrene dish (Förster et al., 1994). *P. sojae* P7076 infection assay was carried out as previously described with some modifications (Jia et al., 2013; Hwu et al., 2017). Briefly, 20 μL 5‰ Tween 20 was dripped on middle of 10-day-old detached leaves, and then transferred 5-day-old fresh mycelial disks approximately 5.5 mm diameter with a 200 μL Eppendorf pipette tip on the middle of detached leaves. Immediately after inoculation, the leaves of soybean were plated in trays covered with plastic wrap to maintain humidity and infected for 48 and 60 h at 25°C in the dark. Infection dead cell was determined by trypan blue staining as described above. Disease resistance was evaluated by measuring the lesion area using Image J software^6^.

## Results

### The Phenotype of *Gmlmm2-1* Mutant Related PCD and ROS Accumulations

**FIGURE 1:**
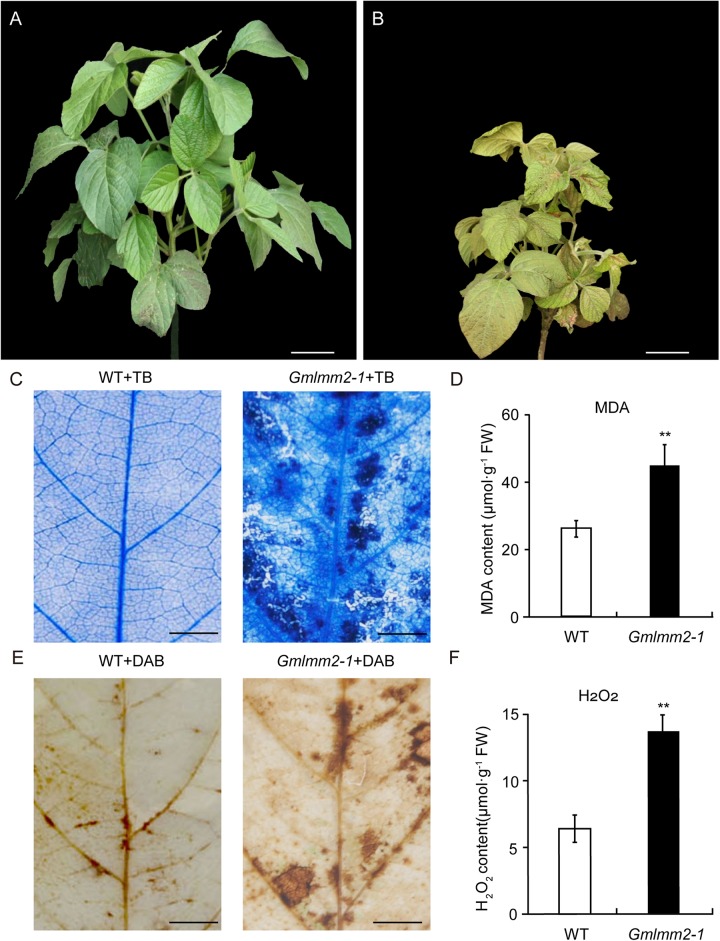
“Williams 82” **(A)** and the *Gmlmm2-1* mutant **(B)** plants. Scale bars = 10 cm. TB staining of leaves of “Williams 82” **(C)** and the *Gmlmm2-1* mutant. Scale bars = 1 cm. **(D)** MDA contents in wild type and mutant leaves. DAB staining of leaves of “Williams 82” **(E)** and the *Gmlmm2-1* mutant. Scale bars = 1 cm. **(F)** H_2_O_2_ contents in wild type and mutant leaves. Asterisks indicate a significant difference by student’s *t*-test (**P < 0.01) and the error bars represent standard deviations.

A lesion mimic mutant was isolated from the EMS-induced “Williams 82” mutant population and named *Gmlmm2-1* (*Glycine max lesion mimic mutant 2-1*). In this mutant, small chlorotic spots were initially visible along the veins of fully expanded leaves, before spreading gradually over the entire leaf. Older leaves of mutant with irregular brown spots began to shrink and senesce rapidly. The adult leaf of mutant was yellow compared with that of “Williams 82” (Figures 1A,B). In addition to necrotic spots, the number of nodules, hundred-grain weight, number of grains per plant, and plant height were reduced in the *Gmlmm2-1* mutant (Supplementary Figure S1).

To examine cell membrane damage situation of leaves, we performed a leaf trypan blue (TB) staining assay of both wild type and mutant at V2 stage (Figure 1C). After trypan blue staining, numerous dark blue spots appeared at the lesion sites on the *Gmlmm2-1* mutant leaves; whereas the surrounding cells in the *Gmlmm2-1* mutant and “Williams 82” cells were healthy (Figure 1C). The content of MDA in the *Gmlmm2-1* mutant was 44.75 ± 6.42 μmol/g, which was 1.70-fold higher than that of “Williams 82” (Figure 1D). The increased level of MDA suggested that lipid peroxidation occurred in the *Gmlmm2-1* mutant. The results of these experiments suggested that PCD occurred during lesion formation.

We subsequently performed DAB staining and observed red-brown polymer deposition in necrotic areas, indicating the generation of excess H_2_O_2_ (Figure 1E). The H_2_O_2_ level in the *Gmlmm2-1* mutant leaves was higher than that in “Williams 82” leaves (Figure 1F), which was consistent with the DAB staining results. These findings indicated that ROS accumulation in cells was responsible for cell death and the visible necrosis in leaves.

### Map-Based Cloning of *GmLMM2*

**FIGURE 2:**
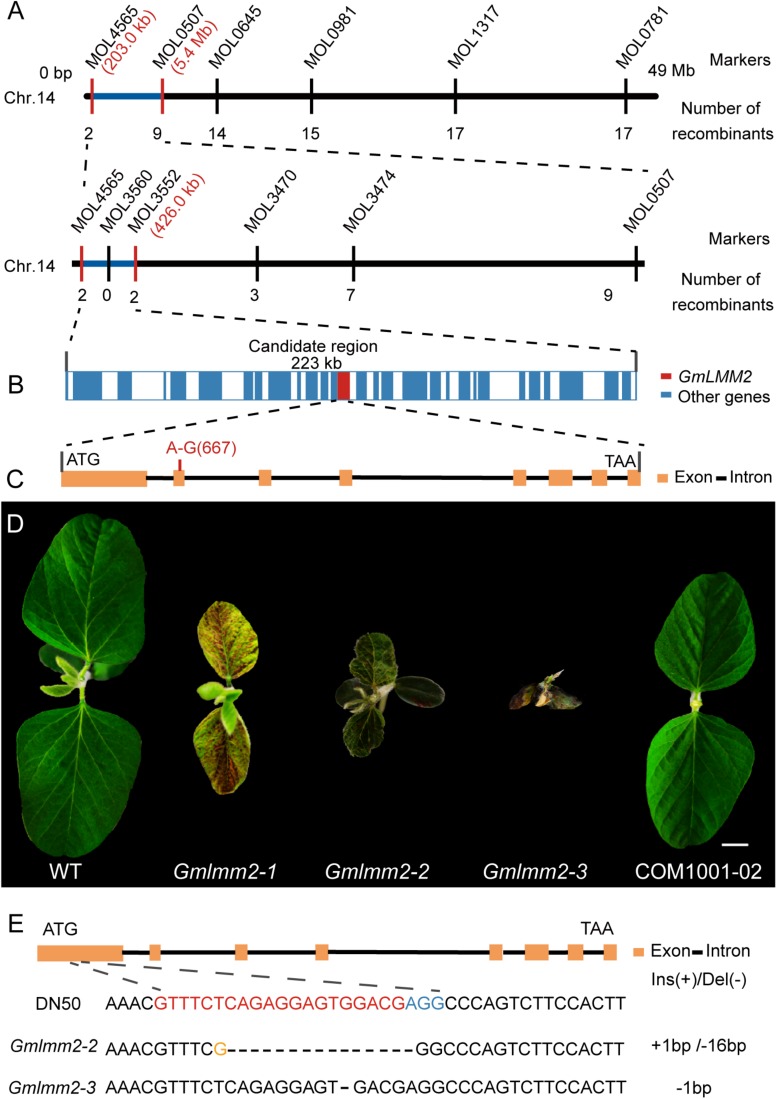
**(A)** Map-based cloning of the *GmLMM2* locus. The numbers under INDELs markers indicate the number of recombinants. **(B)** Diagram of gene distribution in 233kb region harbored *GmLMM2* gene. **(C)** Schematic diagram shows the structure of *Glyma.14G003200*. The red line denotes a A to G substitution in the second exon of *GmLMM2*. **(D)** Phenotypes of “Williams 82,” CRISPR/Cas9 plants (*Gmlmm2-2* and *Gmlmm2-3*), complemented the *Gmlmm2-1* mutant transgenic plant (COM1001-02). Scale bar = 1 cm. **(E)** sgRNA target sequences of “DongNong50,” *Gmlmm2-2* and *Gmlmm2-3*. The sgRNA target sequence is shown in red and the protospacer-adjacent motif (PAM) site in blue. The yellow colored ‘G’ indicates an insertion.

To map the location of *GmLMM2*, we crossed *Gmlmm2-1* mutant with “Hedou 12,” and the F_1_ plants exhibited the wild-type phenotype. Genetic analysis of the F_2_ population revealed that the necrotic phenotype of the *Gmlmm2-1* mutant was controlled by a single recessive nuclear gene (WT: mutant = 618:195). The F_2_ population was used to identify the *GmLMM2* locus. A total of 107 INDEL markers covering all 20 chromosomes were used for preliminary mapping, and the mapping result showed that *GmLMM2* was restricted to the top of Chromosome 14 (Figure 2A). To fine-map the *GmLMM2* locus, we developed four INDEL markers, MOL3470, MOL3552, MOL3560, and MOL4565; the *GmLMM2* locus was further narrowed down to a 223 kb region between 203,085 and 426,404 bp on chromosome 14 (Chr 14) containing 32 annotated genes (Figures 2A,B). To identify the causative mutation, the *Gmlmm2-1* mutant was re-sequenced with a depth of approximately 30 × using illumine Hiseq2000. We identified a A_–667_ to G_–667_ transition at the second exon of *Glyma.14G003200* (Figure 2C), which caused a nonsynonymous substitution of Tyr_–192_ to Cys_–192_ in the predicted protein. No other mutations were discovered among the 32 genes in the candidate *GmLMM2* genomic region (Figure 2B and Supplementary Table S2).

A complementary vector (pFL1002, Supplementary Figure S6) was transformed into the *Gmlmm2-1* mutant. Basta-resistance progenies of the transformed the *Gmlmm2-1* mutant had a wild type phenotype in the T_2_ COM1001-02, COM1001-09, and COM1001-12 populations (Figure 2D, Supplementary Figure S3). Furthermore, we constructed a CRISPR/Cas9 expression vector (pFL1003, Supplementary Figure S7), and the construct was transformed into soybean DN50. Five heterozygous mutants were found in the T_0_ plants, and two independent homozygous knock-out lines, named *Gmlmm2-2* and *Gmlmm2-3*, were found in T_1_ population (Figure 2D). The *Gmlmm2-2* mutant contains a 16 bp deletion corresponding to CDS region of *GmLMM2* gene from 200 bp to 215 bp, and a 1 bp insertion in above region. The *Gmlmm2-2* mutant caused five amino acids deletion from 67th to 71th and one amino acid substitute (Glu to Arg) at 72th of GmLMM2 (Figure 2E). The *Gmlmm2-3* mutant has a 1 bp deletion at 210 bp of CDS and caused a frameshift mutation to a truncated protein with 85 aa (Figure 2E). The *Gmlmm2-2* and *Gmlmm2-3* mutant variedly phenocopied cell death on leaf compared with the *Gmlmm2-1* mutant. The *Gmlmm2-2* and *Gmlmm2-3* mutant also caused a lesion mimic phenotype in the stem, which was not observed in the *Gmlmm2-1* mutant (Supplementary Figure S4). In addition, the *Gmlmm2-2* and *Gmlmm2-3* mutant exhibited pleiotropic phenotypes, including slow growth, dwarfing, and delayed development during vegetative stages, and the *Gmlmm2-3* mutant was a seedling lethal mutant (Figure 2D). These data suggested the *Gmlmm2-2* and *Gmlmm2-3* mutants are strong alleles, whereas the *Gmlmm2-1* mutant is a weak allele, and that complete loss of *GmLMM2* function strongly affects soybean growth and development, and regulates cell death.

### *GmLMM2* Is a Single Copy Gene in the Soybean Genome

**FIGURE 3:**
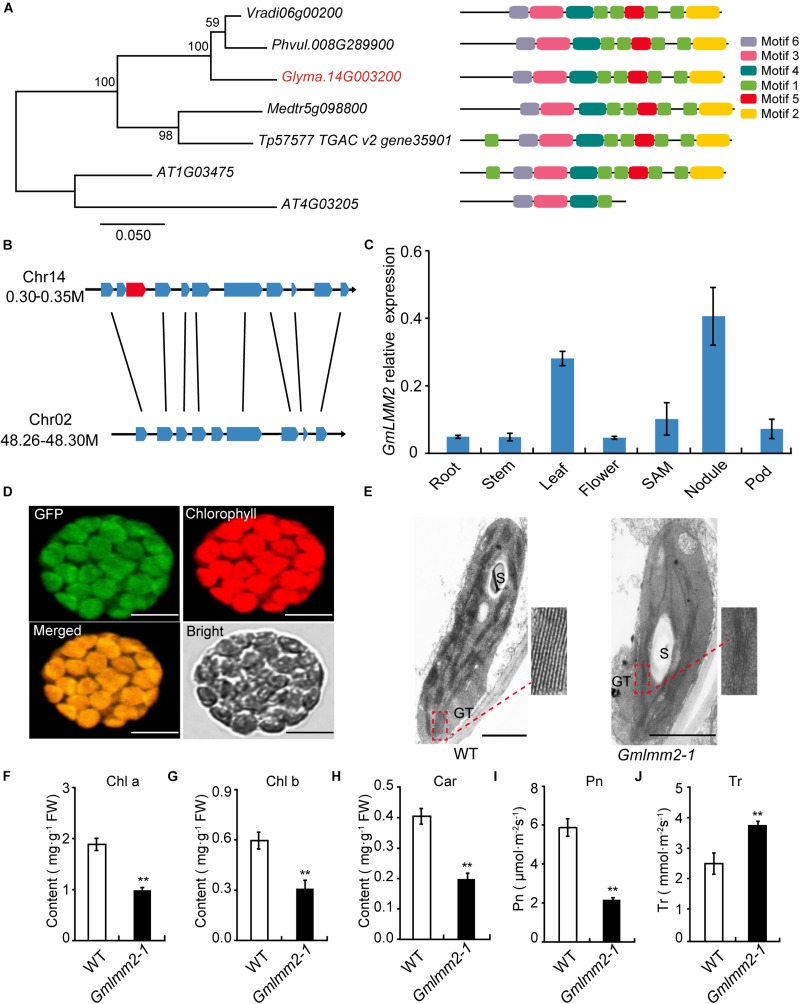
**(A)** Phylogenetic tree of CPOs/HemF in soybean and other species. Seven CPOs/HemF proteins from six species, including *Vigna radiate* (*Vradi06g00200*), *Phaseolus vulgaris* (*Phvul.008G289900*), *Glycine max* (*Glyma.14G003200*), *Medicago truncatula* (*Medtr5g098800*), *Trifolium pretense* (*Tp57577 TGAC v2 gene35901*), and *Arabidopsis thaliana* (*AT1G03475*; *AT4G03205*). **(B)** Synteny diagram plot of the genes surrounding the *GmLMM2* gene. **(C)** Expression pattern of *GmLMM2* in different tissues of “Williams 82” (WT). **(D)** GmLMM2 localized to the chloroplasts. GFP, GFP signal of GmLMM2; Chlorophyll, chlorophyll auto fluorescence; Merged, merged image of GFP and chlorophyll. Scale bars = 10 μm. **(E)** Chloroplast structure of “Williams 82” (WT) and the *Gmlmm2-1* mutant. S, starch granule; GT, granal thylakoid. Scale bars = 1 μm. **(F–J)** Pigment contents and photosynthesis parameters of “Williams 82” and the *Gmlmm2-1* mutant. **(F)** Chl a, chlorophyll a; **(G)** Chl b, chlorophyll b; **(H)** Car, carotenoid; **(I)** Pn, photosynthetic rate; **(J)** Tr, transpiration rate. Asterisks indications are same as Figure 1.

The open-reading frame (ORF) of GmLMM2 is 1158 bp in length, and the deduced protein contains 385 amino acids (Supplementary Figure S5). Phylogenetic analysis showed that GmLMM2 is more closely related to *Phaseolus vulgaris* and *Vigna radiat*e homologs, which constitute an isolated branch in the phylogenetic tree. The identities of *GmLMM2* and the *P. vulgaris* homolog are up to 91%. The results of MEME analysis showed that CPOs contain six conserved motifs (Figure 3A).

To find the homologs of *GmLMM2* gene, we identified the 41.6 kb syntenic block on chromosome 2 from 48,264,660 bp to 48,306,267 bp, which is homologous to a 48.9 kp region to GmLMM2 located from 300,925 bp to 349,856 bp on chromosome 14 (Figure 3B, Supplementary Table S3). They shared 8 holomgous genes between these two regions. However, the putative paralog of *GmLMM2* was absent from the syntenic block on chromosome 2 between *Glyma.02G309600* and *Glyma.02G309700* (Figure 3B). Thus, GmLMM2 is more like a single copy gene in the soybean genome.

We examined *GmLMM2* expression in the root, stem, leaf, flower, nodule, SAM, and pod via quantitative real-time PCR. *GmLMM2* was expressed in all organs and tissues tested (Figure 3C). The highest expression levels were detected in leaf and nodule, which may explain why the *GmLMM2* mutation severely affected the growth of those tissues.

### The *Gmlmm2-1* Mutant Affected Chloroplast Development and Photosynthesis

A chloroplast transit peptide in the first 40 amino acids of GmLMM2 was predicted by TargetP and ChloroP software; and GmLMM2 may be a chloroplast protein. To confirm its subcellular location, a GmLMM2-GFP fusion protein was transiently expressed in Arabidopsis protoplasts. GFP fluorescence only overlapped with the chlorophyll auto fluorescence signal (Figure 3D), which indicated that the GmLMM2 protein is localized to the chloroplast.

To confirm whether *GmLMM2* is associated with chloroplast development, we examined the ultrastructure of plastids in 20-day-old seedlings in “Williams 82” and the *Gmlmm2-1* mutant. The *Gmlmm2-1* mutant contained fewer granal thylakoid membranes compared with the “Williams 82” leaves (Figure 3E). The abnormal chloroplast development in the *Gmlmm2-1* mutant may related to the decreased pigment contents. The contents of Chl a, Chl b, and Car in the *Gmlmm2-1* mutant were approximately 51, 50, and 40% of those in “Williams 82,” respectively (Figures 3F–H). The decreased chlorophyll contents also affected photosynthesis. We found that the net photosynthetic rate of “Williams 82” was 5.93 ± 0.46 μmol CO_2_ m^–2^s^–1^, which was 2.72-fold higher than that of the *Gmlmm2-1* mutant (Figure 3I). The transpiration rate of the *Gmlmm2-1* mutant was 3.75 ± 0.12 mmol H_2_O m^–2^s^–1^, which was 1.49-fold higher than that of “Williams 82” (Figure 3J). However, maximum chlorophyll fluorescence (Fv/Fm) and initial fluorescence ratio (Fv/F_0_) did not vary between the *Gmlmm2-1* mutant and “Williams 82” (Supplementary Figure S2).

### The Tetrapyrrole Biosynthesis Pathway Is Disturbed in the *Gmlmm2-1* Mutant

**FIGURE 4:**
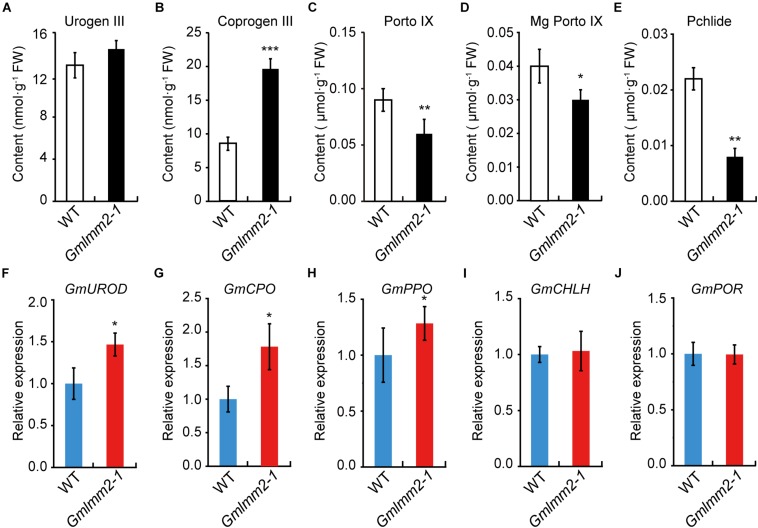
**(A–E)** Contents of metabolic intermediates in young leaves of wild type and mutant. **(F–J)** Expression levels of some enzymes in tetrapyrrole biosynthesis of wild type and mutant. Asterisks indicate a significant difference by student’s *t*-test (*P < 0.05, **P < 0.01, ***P < 0.001) and the error bars represent standard deviations.

To further understand the impact of the *GmLMM2* mutation on the tetrapyrrole biosynthesis pathway, the contents of some metabolic intermediates of tetrapyrrole biosynthesis were examined. No significant difference in Uroporphyrinogen III (Urogen III) levels was observed between wild type and mutant (Figure 4A). Whereas, the Coproporphyrinogen III (Coprogen III) level in the *Gmlmm2-1* mutant was two-fold higher than that in wild type (Figure 4B). The levels of Protoporphyrinogen-IX (Proto IX), Mg-protoporphyrinogen-IX (Mg-proto IX), and Pchlide in the *Gmlmm2-1* mutant were significantly lower than those in the leaves of “Williams 82” (Figures 4C–E). Therefore, light-dependent cell death in the *Gmlmm2-1* mutant may be attributed to the accumulation of Coprogen III.

We further analyzed the expressions of some enzymes related above intermediates, including uroporphyrinogen III decarboxylase (*GmUROD*, *Glyma.18G021500*); coproporphyrinogen III oxidase (*GmCPO*, *Glyma.14G003200*), protoporphyrinogen oxidase (*GmPPO*, *Glyma.10G138600*); Mg-chelatase subunit H (*GmCHLH*, *Glyma.10G097800*) and NADPH: Pchlide oxidoreductase (*GmPOR*, *Glyma.12G222200*). The transcript levels of *GmUROD*, *GmCPO*, and *GmPPO* were slightly elevated, but the transcript levels of *GmCHLH* and *GmPOR* remained substantially unchanged (Figures 4F–J). The expression raise of *GmUROD* and *GmCPO* might response to their substrates increasing in mutant plant.

### The Cell Death Phenotype of the *Gmlmm2-1* Mutant Is Light-Dependent

**FIGURE 5:**
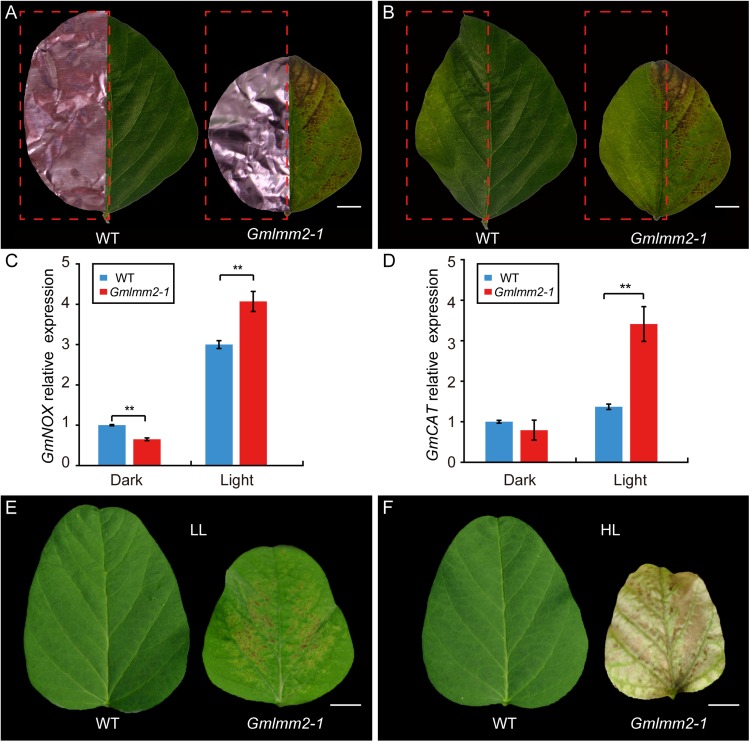
Light avoidance assay in leaves of “Williams 82” and the *Gmlmm2-1* mutant before **(A)** and after **(B)** removing the shading foils. Scale bars = 0.5 cm. Comparison of expression levels of *GmNOX* **(C)** and *GmCAT* **(D)** between shaded (Dark) and un-shaded (Light) half leaflet. Phenotype of leaf growing under **(E)** lower intensity light (LL) and **(F)** high intensity light (HL). Scale bars = 1 cm. Asterisks’ indications are same as Figure 1.

To confirm whether the formation of lesions in the *Gmlmm2-1* mutant leaves was light-dependent, half of the leaflets before lesion emergence were covered by aluminum foil, while the other half of the leaflets were exposed to light. Five-days later, no lesion had formed on the non-shaded or shaded leaflets of “Williams 82.” However, irregular brown lesions appeared on the light-exposed leaflets of the *Gmlmm2-1* mutant, but not on the shaded part leaflets of the *Gmlmm2-1* mutant (Figures 5A,B).

NADPH oxidase (NOX) is related to ROS homeostasis, and catalase (CAT) is a ROS-scavenging gene (Lee et al., 2005, 2018). To determine the relationship of ROS and light condition, we compared expression of *GmNOX* and *GmCAT* of half leaflet with light covering. The transcript level of *GmNOX* in the *Gmlmm2-1* mutant was 1.35-fold higher than that in the “Williams 82” under light. The expression of *GmCAT* in the *Gmlmm2-1* mutant was 1.65-fold higher than that in the “Williams 82” under light. There was no significant difference in the expression of *GmCAT* between the *Gmlmm2-1* mutant and “Williams 82” in the aluminum foil-covered leaves (Figures 5C,D). The results demonstrated that the *GmLMM2* defect disrupts ROS homeostasis in the *Gmlmm2-1* mutant, and that these processes are light-dependent.

The effect of light intensity on the lesion induction was also observed under different light condition. We planted “Williams 82” and *Gmlmm2-1* mutant under high (20,000 Lux) and low (7,500 Lux) intensity light, respectively. We found that the higher intensity light rapidly and broadly induced severe necrosis and development, and chlorophyll deficiency compared with low intensity light (Figures 5E,F). These results suggested that light is a critical causal factor for lesion induction in the *Gmlmm2-1* mutant and light intensity is correlated with the degree of cell death.

### Disease Reaction of the *Gmlmm2-1* Mutant to *Phytophthora sojae* P7076

**FIGURE 6:**
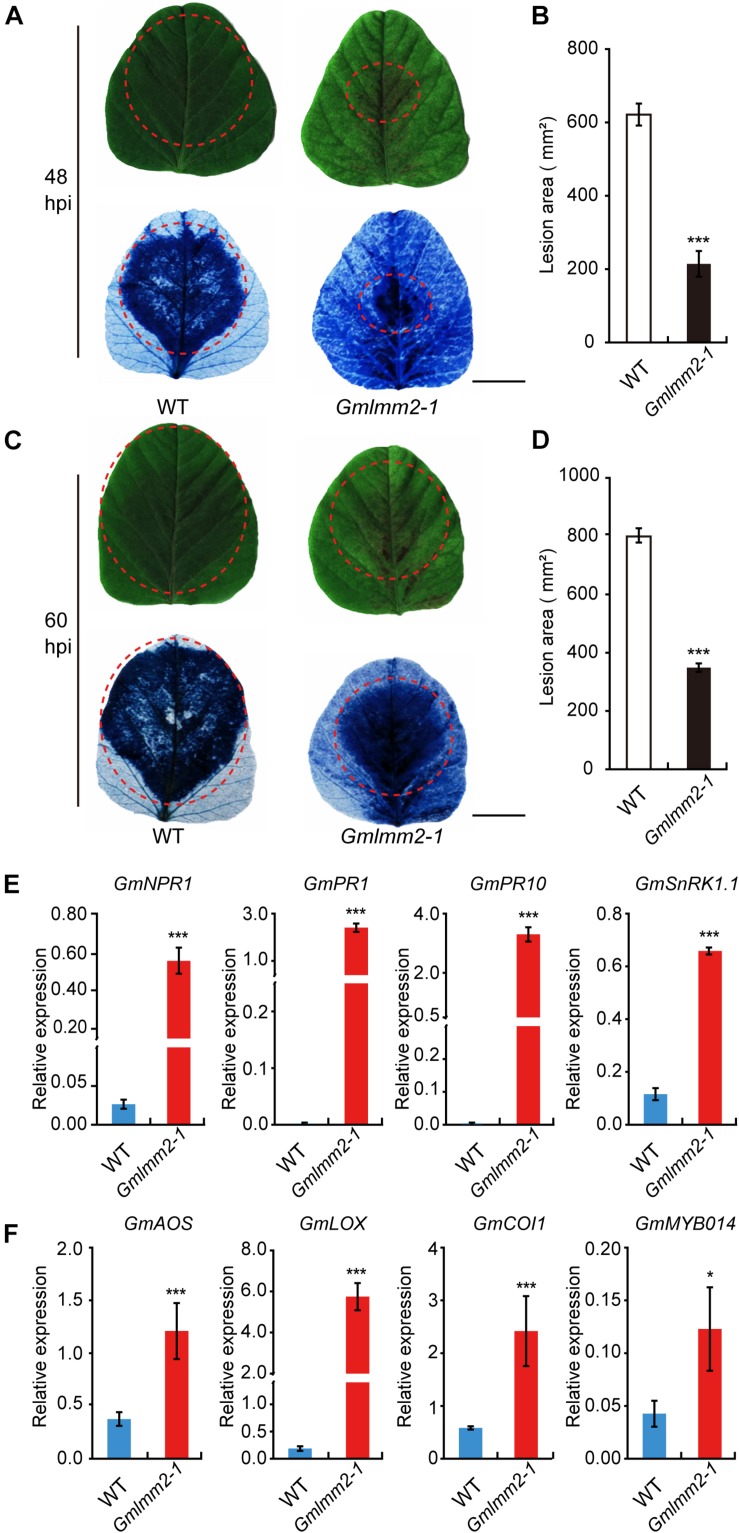
Up-regulation of resistance-related genes and enhanced pathogen resistance. **(A–D)** The infection phenotypes of “Williams82” (WT) and the *Gmlmm2-1* mutant at 48 and 60 hpi with *Phytophthora sojae* stain P7076. Trypan blue staining demonstrated that infection levels were similar to those of the leaves shown above. Scale bars = 1 cm. The sizes of the lesions were statistically analyzed corresponding to **(A–C)**. **(E,F)** The expression levels of SA-signaling and JA-signaling genes in “Williams82” (WT) and the *Gmlmm2-1* mutant. The data shown are the mean values of three biological replicates and the error bars represent the standard deviation (SD). Data are presented as fold difference from the WT control after normalizing to the control gene *GmActin11*. Relative expression levels are presented as means ± SD. Data are from three biological replicates. *P < 0.05, **P < 0.01, ***P < 0.001 (*t*-test).

The previously study of lesion mimic mutant suggested that the *Gmlmm2-1* mutant may enhanced resistance to pathogen infection. To confirm this hypothesis, the fully expanded 10-day-old true leaves of “Williams 82” and the *Gmlmm2-1* mutant were inoculated with *P. sojae* P7076. Visible lesions were observed on the leaves of “Williams 82” and the *Gmlmm2-1* mutant at 48 h post-inoculation (48 hpi) (Figure 6A). We measured the infectious area by TB staining, the infectious area in *Gmlmm2-1* mutant reduced to 34.5 and 43.7%, compared with “Williams 82” at 48 and 60 hpi separately (Figures 6B–D). These results suggested that mutation of *GmLMM2* inhibits the invasive of *P. sojae* P7076 and the *Gmlmm2-1 mutant* enhanced resistance to *P. sojae* P7076.

To test whether spontaneous lesion formation in mutant correlated with the expression of resistance-related genes, we monitored the transcript levels of these genes in “Williams 82” and the *Gmlmm2-1* mutant. SA-signaling genes (*GmNPR1*, *GmPR1*, *GmPR10*, and *GmSnRK1.1*) were more highly expressed in the *Gmlmm2-1* mutant compared with those in “Williams 82” (Figure 6E). The transcript level of *GmSnRK* in the *Gmlmm2-1* mutant was 5.5-fold higher than that in “Williams 82.” The expression of *GmPR10* in the *Gmlmm2-1* mutant was 1373.3-fold higher than that in “Williams 82.” As shown in Figure 6F, these JA-signaling genes (*GmAOS*, *GmLOX*, *GmCOI1*, and *GmMYB014*) were activated at different levels in the *Gmlmm2-1* mutant. The expression of *GmCOI1* in the *Gmlmm2-1* mutant was 4.17-fold higher than that in “Williams 82.” These results indicated that the expressions of defense-related genes are up-regulated during lesion development.

## Discussion

### Light-Dependent Cell Death Caused by ROS Accumulation in the *Gmlmm2-1* Mutant

Coproporphyrinogen III Oxidase (CPO) catalyzes the coproporphyrinogen-III to yield protoporphyrinogen-IX in tetrapyrrole biosynthesis. Defects in CPO have been reported in *Nicotiana tabacum*, *Arabidopsis*, and rice (Kruse et al., 1995; Ishikawa et al., 2001; Sun et al., 2011; Wang et al., 2015). This study is the first report to demonstration the function of CPO in soybean. Many *lmms* mutants exhibit light-dependent cell death, accompanied by the upregulation of ROS-related genes (Sathe et al., 2019). In our study, the *GmLMM2* defect in the *Gmlmm2-1* mutant also induced a light-dependent lesion mimic phenotype, and higher light intensity led to severe necrosis and chlorophyll deficiency (Figures 5A,B,E,F). Trypan blue and DAB staining assays showed that cell death in the *Gmlmm2-1* mutant was caused by abnormal H_2_O_2_ accumulation (Figure 1). The up-regulation of *GmNOX* led to ROS accumulation in the illuminated region of the *Gmlmm2-1* mutant, and not in the dark region (Figure 5C). As a large amount of H_2_O_2_ accumulated in the illuminated region of the *Gmlmm2-1* mutant, expression of the genes encoding H_2_O_2_-scavenging enzymes, such as *GmCAT*, was significantly upregulated (Figure 5D). ROS accumulation can trigger the expression of resistance-related genes, such as PR genes; therefore, many *lmm*s have enhanced resistance to pathogens (Dangl and Jones, 2001; Guo et al., 2013; Wang et al., 2015; Lv et al., 2019).

### Defense Response Is Induced in the *Gmlmm2-1* Mutant

Resistance-related genes may be activated in *lmms* and contribute to enhanced pathogen resistance (Guo et al., 2013; Wang et al., 2015). In the present study, the expressions of important genes involved in JA and SA signaling pathways were upregulated in the *Gmlmm2-1* mutant and the *Gmlmm2-1* mutant displayed enhanced resistance to *P. sojae* P7076. The SA pathway has been implicated in conferring resistance to biotrophic pathogens, such as *Pseudomonas syringae* and *Hyaloperonospora arabidopsidis*, and the JA pathway in resistance to necrotrophic pathogens, such as *Alternaria brassicicola* and *Botrytis cinerea* (Penninckx et al., 1998; Pieterse and Van Loon, 1999; Glazebrook, 2005; Spoel et al., 2007). The induction of marker genes involved in the two defense signaling pathways suggested that the *Gmlmm2-1* mutant may have acquired a broad spectrum of resistance to multiple pathogens.

In summary, the *Gmlmm2-1* mutant exhibits light-induced lesions. The identification of *GmLMM2* improves our understanding of the molecular mechanism underlying cell death and the defense response in soybean. In addition, it helps us to further understand the complex regulation of the tetrapyrrole biosynthesis pathway in soybean.

## Data Availability Statement

Sequences were deposited at the National Center for Biotechnology Information (NCBI) under the accession number SRP149750.

## Author Contributions

SY and XZF conceived and designed the study. JM, DW, and XXF performed the experiments and wrote the manuscript. JM, KT, and XXF modified the images. KT analyzed the sequencing data. JM, SY, and XZF discussed the results and prepared the manuscript. All authors read and approved the manuscript.

## Conflict of Interest

The authors declare that the research was conducted in the absence of any commercial or financial relationships that could be construed as a potential conflict of interest.

## Footnotes

1 http://www.phytozome.net

2 https://www.megasoftware.net/

3 http://meme-suite.org/

4 http://cbi.hzau.edu.cn/crispr

5 http://www.genome.arizona.edu/crispr/index.html

6 https://imagej.en.softonic.com
