# Corrigendum: Genetic Mapping of a Light-Dependent Lesion Mimic Mutant Reveals the Function of Coproporphyrinogen III Oxidase Homolog in Soybean

**DOI:** 10.3389/fpls.2020.00893

**Published:** 2020-07-10

**Authors:** Jingjing Ma, Suxin Yang, Dongmei Wang, Kuanqiang Tang, Xing Xing Feng, Xian Zhong Feng

**Affiliations:** ^1^Key Laboratory of Soybean Molecular Design Breeding, Northeast Institute of Geography and Agroecology, The Innovative Academy of Seed Design, Chinese Academy of Sciences, Changchun, China; ^2^University of Chinese Academy of Sciences, Beijing, China

**Keywords:** *Glycine max*, *lesion mimic mutant 2*, tetrapyrrole biosynthesis, coproporphyrinogen III oxidase, pathogen resistance

In the original article, the incorrect names were indicated as the last name for the three authors for Fang et al. 2017. They should be “Hwu, F. Y., Lai, M. W., and Liou, R. F.” and so the reference should now change to Hwu et al. 2017.

The authors apologize for this error and state that this does not change the scientific conclusions of the article in any way. The original article has been updated.

