# Supplementary material for: Genetic Mapping of a Light-Dependent Lesion Mimic Mutant Reveals the Function of Coproporphyrinogen III Oxidase Homolog in Soybean
Source: Front Plant Sci. 2020 May 8;11:557. doi: 10.3389/fpls.2020.00557 (PMC7227399; doi:10.3389/fpls.2020.00557)
Supplement: Supplementary file 1 [file Presentation_1.pdf]

SUPPLEMENTARY MATERIAL:

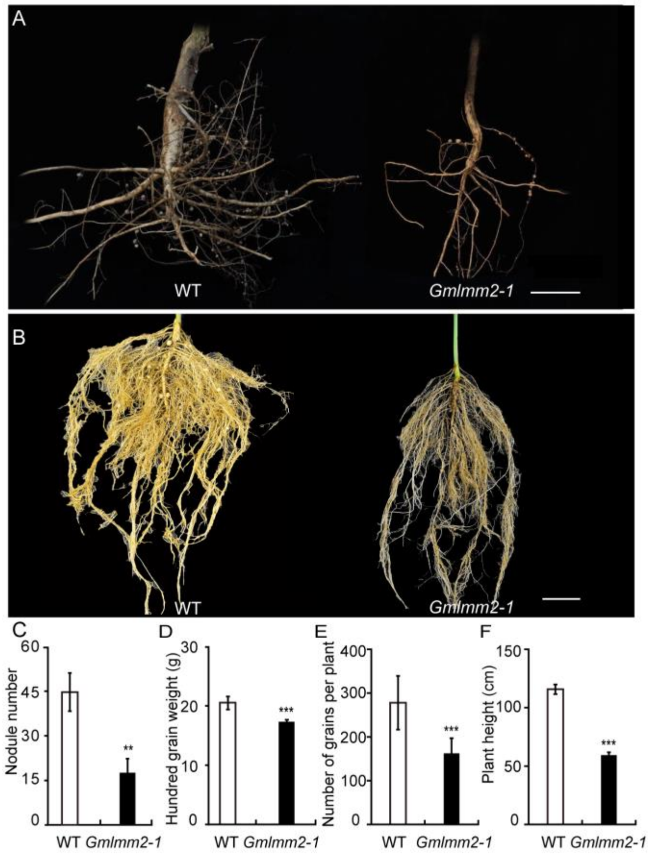

Figure S1 Nodule phenotype and yield indices. (A) Nodule phenotype in the fields. Scale bar = 5 cm. (B) Nodule phenotype in growth chamber. Scale bar = 3 cm. (C) Nodule number in the fields. (D) Hundred grain weight in the fields. (E) Number of grains per plant in the field. (F) Plant height oin the fields. The results are presented as the means ± standard deviation, which were calculated from 10 different plants. Asterisks indicate a significant difference by student's t-test (**P<0.01,*** p<0.001).

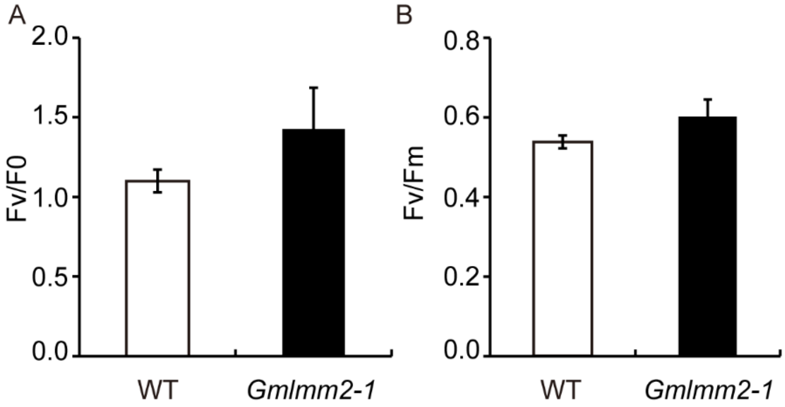

Figure S2 Chlorophyll fluorescence parameters. Fv/Fm (maximum quantum efficiency of photosystem II) and Fv/F_0 (maximum primary yield of photochemistry of PSII) for the 'Williams 82' and the *Gmlmm2-1* mutant. Data are from three biological replicates. The results are presented as the means ± standard deviation.

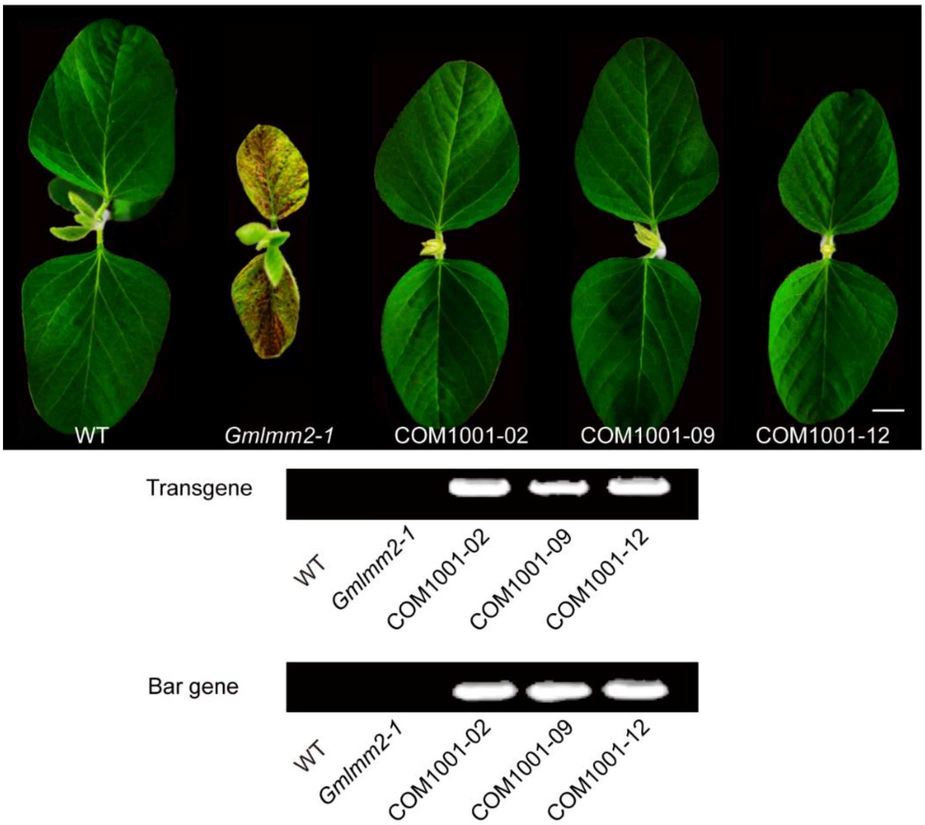

Figure S3 The phenotype of complemented transgenic lines. Genotyping of *GmLMM2* in 'Williams 82' (WT), the *Gmlmm2-1* mutant, and the complemented transgenic lines (COM1001-02, COM1001-09, and COM1001-12).

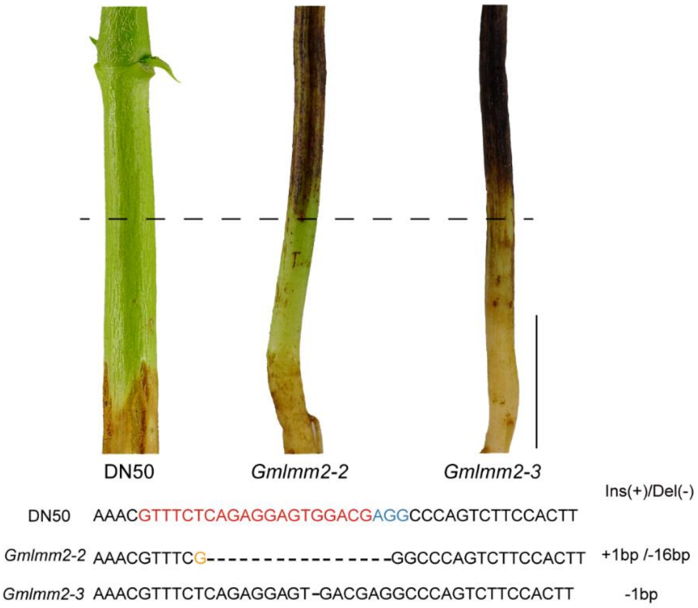

Figure S4 The phenotype and sgRNA target sequences of the CRISPR/Cas9 lines. Scale bar = 1 cm. The sgRNA target sequence is in red and the protospacer-adjacent motif (PAM) site is in blue.

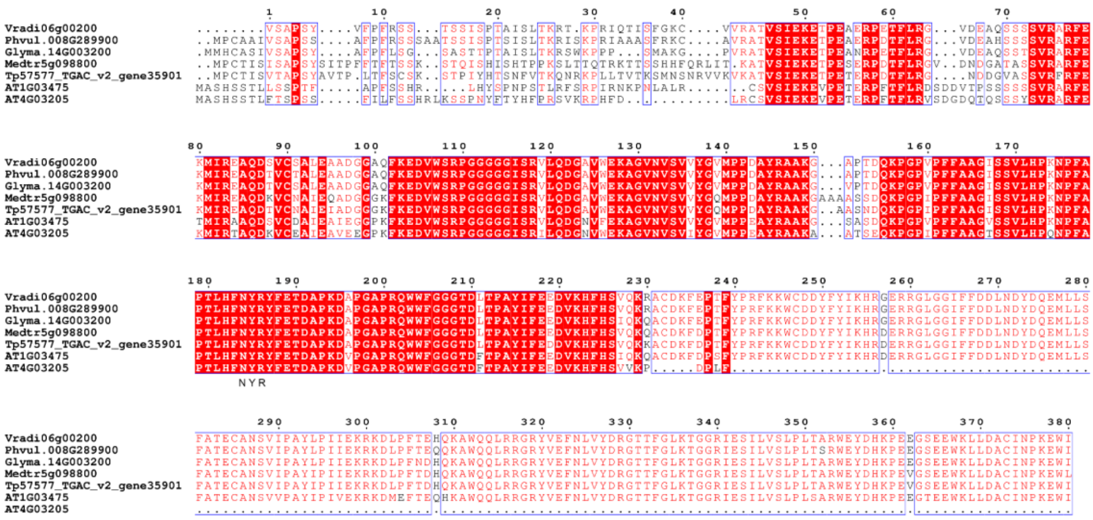

Figure S5 Amino acid sequence alignment of GmLMM2 and its homologues. Comparison of the amino acid sequences of GmLMM2 with that of its homologues from related plant species, including *Vigna radiate* (*Vradi06g00200*), *Phaseolus vulgaris* (*Phvul.008G289900*), *Glycine max* (*Glyma.14g003200*), *Medicago truncatula* (*Medtr5g098800*), *Trifolium pretense* (*Tp57577 TGAC v2 gene35901*), *and Arabidopsis thaliana* (*AT1G03475; AT4G03205*)revealed a conserved tyrosine residue at the position 192. The black arrow indicates the location of the substrate-binding site.

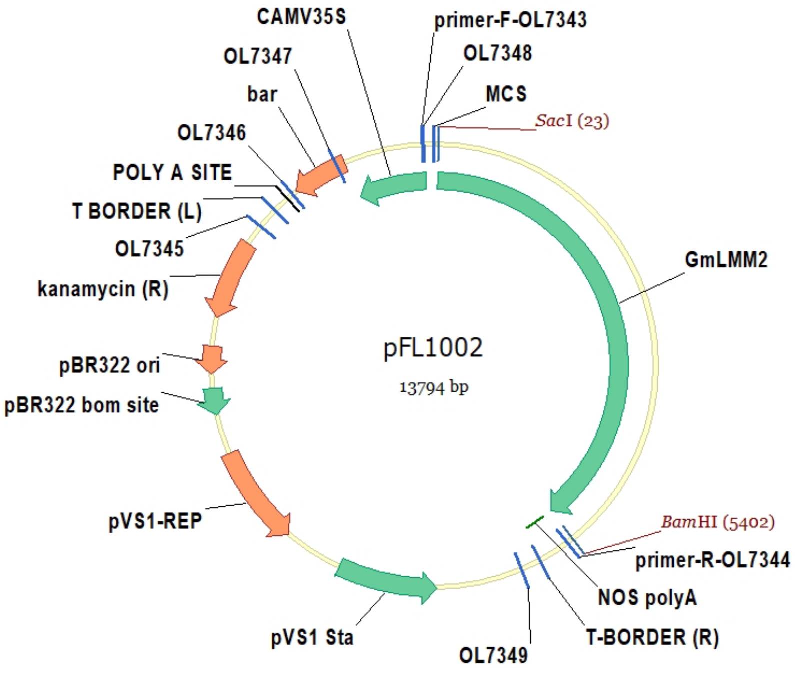

Figure S6 Diagram of plasmid pFL1002.

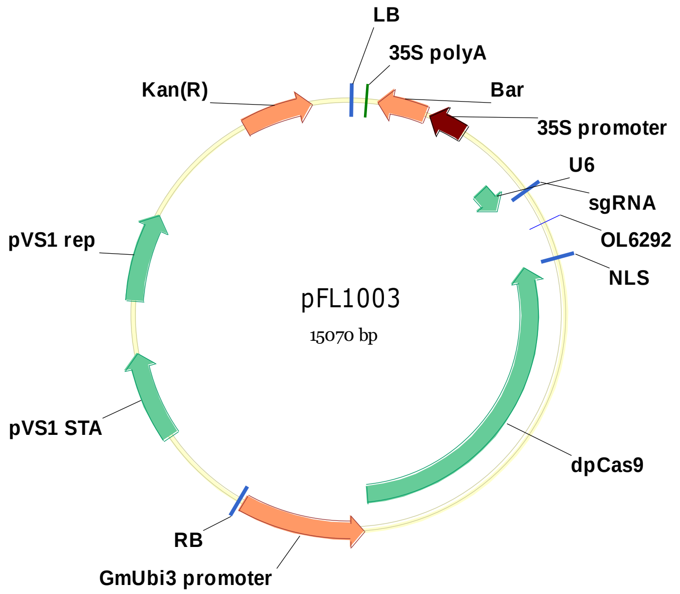

Figure S7 Diagram of plasmid pFL1003.

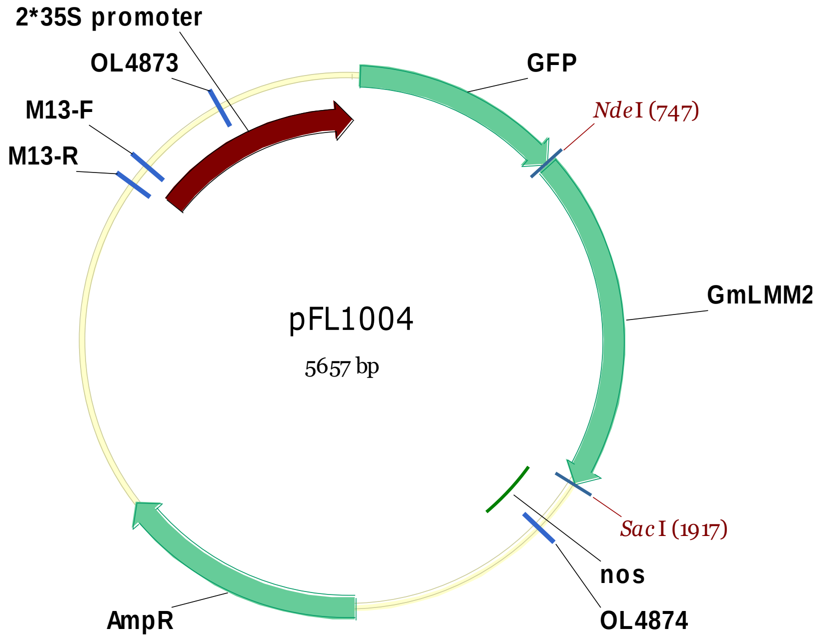

Figure S8 Diagram of plasmid pFL1004.

Table S1 Primers used in this study

Primer name	Sequence (5'-3')	Application
MOL0507F	GGGACGGGTGGGAATTGTAGTTGCTT	mapping
MOL0507R	TAGACAACCTTTTCTTGCACCCTCCC	mapping
MOL0645F	GAGCACAAACCATTAGGCTTCAA	mapping
MOL0645R	TGGGGGAGATATTGATTGATAGATG	mapping
MOL0781F	CAAAGGCAAGAAGGATCAAAGCA	mapping
MOL0781R	GAAAAATCACATCAAGCCAACTC	mapping
MOL3462F	AATAGAGGGAGCGACAACAGGC	fine mapping
MOL3462R	GAAGTCACATCAGACGTTCAAACAC	fine mapping
MOL3470F	CTGAATGGACTTCCCAAAGGCA	fine mapping
MOL3470R	GGAGCAAACCTAACAGAGAAACAAG	fine mapping
MOL3474F	ACCCCCTTACCACCCTATTGATAC	mapping
MOL3474R	GCTTGAGGCTTCTGTTCAAATTG	mapping
MOL3552F	TTCCTATCACATCCTATAATCTATC	fine mapping
MOL3552R	TTACAACCATCCCAAACACGCT	fine mapping
MOL3476F	CTATCTCCATCTGCATCTGCTACG	fine mapping
MOL3476R	AAGAGAACTGGTTAGAGAGGAAAGG	fine mapping
OL8376F	CGCGGATCCGTCAAAGTCCTTACATTTACCTACC	pFL1002
OL8376F	CGCGGATCCTTAGATCCATTCCTTGGGGTTG	pFL1002
OL7022F	ATTGTTTCTCAGAGGAGTGGACG	pFL1003
OL7022R	AACCGTCCACTCCTCTGAGAAAC	pFL1003
OL7674F	GGAATTCCATATGATGATGCATTGTGCGAGC	pFL1004
OL7674R	CAAGAGCTCTTAGATCCATTCCTTGGGGTTGAT	pFL1004
OL7343	GCAAGCTGCTCTAGCCAATAC	pCAMBIA3301
OL7670F	ATGACAGCGACCACGCTCTTGAAG	Bar gene
OL7670R	CTGCACCATCGTCAACCACTACATC	Bar gene
OL1016F	CGGTGGTTCTATCTTGGCATC	RT-qPCR(*GmActin11*)
OL1016R	GTCTTTCGCTTCAATAACCCTA	RT-qPCR(*GmActin11*)
MOL3888F	ATTGTCTCGGCTCCGTCCTA	RT-qPCR(*GmLMM2*)
MOL3888R	ATAGAAACGGTGGCTCTGACTG	RT-qPCR(*GmLMM2*)
MOL3892F	CTGCTGGTGAACCTCTTTTGCTA	RT-qPCR(*GmUROD*)
MOL3892R	CATTTTCAGATCTTTCACGGAATG	RT-qPCR(*GmUROD*)
MOL3900F	TGAGGAATCGGTTGAAGAGTTTG	RT-qPCR(*GmPPO*)
MOL3900R	TTTCCCGAATGCTGCTTTCATACT	RT-qPCR(*GmPPO*)
OL9148F	AGCCTCTCCAGCAGTTACCAAG	RT-qPCR(*GmPOR*)
OL9148R	ATTACATGCCATTTTCCCGTCTC	RT-qPCR(*GmPOR*)
OL10014F	CTCTCTTCTCTTGCCCAGAGACACC	RT–qPCR(*GmCHLH*)
OL10014R	TGAGTGAAAAGACCATTGCCCATAG	RT-qPCR(*GmCHLH*)
OL10002F	AGGTAGCACTTGCACTCCACAAACT	RT-qPCR(*GmNOX*)
OL10002R	GCTCAGTTCAATTGCAGCTCGGATA	RT-qPCR(*GmNOX*)
OL10004F	TCGATCAATTTGAAAGAACTTATCC	RT-PCR(*GmCAT*)

OL10004R	GAACACATAGTCCACAACAATAGCA	RT-qPCR(*GmCAT*)
OL7636F	ACCGGTAACCTAAGTGGTACAAATG	RT-qPCR(*GmPR1*)
OL7636R	CCACATCCAAGACGCACAGAGT	RT-qPCR(*GmPR1*)
OL7640F	AGTCTCCACTTCCGCCGTATGAT	RT–qPCR(*GmCOI1*)
OL7640R	CGTGTAACCATTCTCCGTCCTTC	RT–qPCR(*GmCOI1*)
OL7642F	GCTTCTCTCTCAGTGCATTCGTAGA	RT–qPCR(*GmNPR1*)
OL7642R	AAGGACTCCACTTCCATTGCATTAG	RT–qPCR(*GmNPR1*)
OL10432F	CTCGAGCCCATCCTCGTGAAATAAT	RT-qPCR (*GmSnRK1.1*)
OL10432R	AACCCACCTACACTTCATGTTGTAG	RT-qPCR (*GmSnRK1.1*)
OL10436F	TAGCATCCACAGCATTGTTTTC	RT-qPCR(*GmPR10*)
OL10436R	CAAGGCAGTGCCCTCAGTTA	RT-qPCR(*GmPR10*)
OL10440F	ACTACTCCTGAGAATAGTAGCACGG	RT-qPCR(*GmMYB014*)
OL10440R	TCAGGGACACTGTAATATTCAGTCA	RT-qPCR(*GmMYB014*)
OL10442F	GGCCCTTGAGAAGTAAATTAGATCC	RT-qPCR(*GmLOX*)
OL10442R	CTGTGCAATAGCCAATTATTTGTGG	RT-qPCR(*GmLOX*)
OL10444F	TCTTCTACGAGTCTTCCGGTCTAGT	RT-qPCR(*GmAOX*)
OL10444R	AGTTAAAGCAGGTGGCGAACAACAG	RT-qPCR(*GmAOX*)

Table S2 Genes within the candidate area

Gene name	Best-hit-arabi-name	arabi-defline
Glyma.14G001700	*AT4G35280*	C2H2-like zinc finger protein
Glyma.14G001800	*AT4G38370*	Phosphoglycerate mutase family protein
Glyma.14G001900	*AT4G35260*	Isocitrate dehydrogenase 1
Glyma.14G002000	*AT4G38380*	MATE efflux family protein
Glyma.14G002100	*AT1G76270*	O-fucosyltransferase family protein
Glyma.14G002200		
Glyma.14G002300	*AT4G35240*	Protein of unknown function (DUF630 and DUF632)
Glyma.14G002400	*AT4G35230*	BR-signaling kinase 1
Glyma.14G002500	*AT3G22250*	UDP-Glycosyltransferase superfamily protein
Glyma.14G002600		
Glyma.14G002700	*AT5G60580*	RING/U-box superfamily protein
Glyma.14G002800	*AT4G39720*	VQ motif-containing protein
Glyma.14G002900	*AT2G32260*	Phosphorylcholine cytidylyltransferase
Glyma.14G003000	*AT3G22150*	Tetratricopeptide repeat (TPR)-like superfamily protein
Glyma.14G003100	*AT1G24290*	AAA-type ATPase family protein
Glyma.14G003200	*AT1G03475*	Coproporphyrinogen III oxidase
Glyma.14G003300	*AT5G11340*	Acyl-CoA N-acyltransferases (NAT) superfamily protein
Glyma.14G003400	*AT1G61520*	Photosystem I light harvesting complex gene 3
Glyma.14G003500	*AT4G12650*	Endomembrane protein 70 protein family
Glyma.14G003600	*AT5G48310*	
Glyma.14G003700	*AT1G57820*	Zinc finger (C3HC4-type RING finger) family protein
Glyma.14G003800	*AT3G13130*	
Glyma.14G003900	*AT5G09950*	Tetratricopeptide repeat (TPR)-like superfamily protein
Glyma.14G004000	*AT2G05310*	
Glyma.14G004100	*AT4G16360*	5\'-AMP-activated protein kinase beta-2 subunit protein
Glyma.14G004200	*AT4G12730*	FASCICLIN-like arabinogalactan 2
Glyma.14G004300	*AT5G47650*	Nudix hydrolase homolog 2
Glyma.14G004400	*AT3G07750*	3\'-5\'-exoribonuclease family protein
Glyma.14G004500	*AT3G07700*	Protein kinase superfamily protein
Glyma.14G004600	*AT5G47720*	Thiolase family protein
Glyma.14G004700	*AT2G29530*	Tim10/DDP family zinc finger protein
Glyma.14G004800	*AT1G31335*	

Table S3 Syntenic genes in chromosome 14 and chromosome 2

Gene ID	Gene ID
Glyma.14G003000	*Glyma.02G308900*
Glyma.14G003100	*Glyma.02G309000*
Glyma.14G003200	*Glyma.02G309100*
Glyma.14G003300	*Glyma.02G309200*
Glyma.14G003400	*Glyma.02G309300*
Glyma.14G003500	*Glyma.02G309400*
Glyma.14G003600	*Glyma.02G309500*
Glyma.14G003700	*Glyma.02G309600*
Glyma.14G003800	*Glyma.02G309700*
Glyma.14G003900	
Glyma.14G004000	
